# Biological activities of derived pigments and polyphenols from the newly recorded alga *Phyllymenia gibbesii*

**DOI:** 10.1038/s41598-024-70825-5

**Published:** 2024-09-11

**Authors:** Nihal G. Shams El-Din, Mohamed S. M. Abd El Hafez, Miral G. Abd El-Wahab, Hassan A. H. Ibrahim

**Affiliations:** 1 National Institute of Oceanography and Fisheries, NIOF, Cairo, Egypt; 2 Faculty of Health Science Technology, Borg Al Arab Technological University, Alexandria, Egypt; 3 Faculty of Technological Industry and Energy, Thebes Technological University, Thebes, Luxor, Egypt; 4 Pharmaceutical and Fermentation Industries Development Centre (PFIDC), City of Scientific Research and Technological Applications (SRTA-City), Borg Al Arab Al Gadida city, Egypt; 5 Microbiology Department, NIOF, Kayet Bay, El-Anfoushy, Alexandria, Egypt

**Keywords:** Biological techniques, Biotechnology, Ecology, Microbiology, Plant sciences

## Abstract

The newly recorded *Phyllymenia gibesii* in the Mediterranean Sea at Alexandria coast of Egypt is regarded as a significant source of bioactive substances and is applied as an antioxidant, anti-inflammatory, and antimicrobial agent. According to the HPLC chromatograms, the acetone extract of *P. gibesii* comprised ten photosynthetic pigments (chlorophyll-a, chlorophyll-d, α-carotene, β-carotene, phycocyanin, allophycocyanin, antheraxanthin, β-cryptoxanthin, lutein, and violaxanthin). Total carotenoids were the dominant class in the pigments’ profile, achieving a concentration of 257 g/g dry weight. The *P. gibbesii* extract had a total content of phenols (146.67 mg/g) and a total content of flavonoids (104.40 mg/g). The capacity of all the investigated biological activities augmented with the concentration of the algal extract. The maximal DPPH scavenging capacity was 81.44%, with an inhibitory concentration (IC_50_) of 9.88 μg/mL. Additionally, the highest ABTS scavenging capacity was 89.62%, recording an IC_50_ of 21.77 μg/mL. The hemolytic activity of *P. gibbesii* attained a maximum capacity of 49.88% with an IC_50_ of 100.25 μg/mL. Data also showed the maximum anti-inflammatory effectiveness at 81.25%, with an IC_50_ of 99.75 μg/mL. Furthermore, the extract exhibited antimicrobial capacity against all reference strains, particularly at high concentrations (0.1 mg/mL), with the greatest effect on *C. albicans* and *E. coli*.

## Introduction

Marine macroalgae comprised three groups, among which the Rhodophyceae (red algae) are the most diversified in the world (circa 6131 species)^1^. Numerous types of red algae serve as a good source of natural substances with significant biological functions involving antimicrobial^2^, anti-inflammatory^2^, antihyperlipidemic^3^, antidiabetic^3^, antitumors^3–5^, and antioxidant activities^3–5^.

The benefits to human health are taken into account when using red algae antioxidants in foods, food supplements, nutraceuticals, and medicines^4^. Red algae have gained significant economic value from these bioproducts, such as agar and carrageenan, making them a valuable commodity from an industrial perspective^4^. However, the concentration and composition of bioactive substances in seaweeds might vary across individuals, depending on their environment, the season, and other factors. Regarding climatic change, the environmental factors affecting intertidal algae are continuously changing. To deal with shifting of environmental conditions, seaweeds involving red algae modified specific adaptive metabolic responses, making them able to withstand and adapt to various abiotic pressures^5^.

Furthermore, the pigments of red algae showed a diverse spectrum of biological functions. As photoautotrophic, they own chlorophyll-a, and chlorophyll-d, and accessory pigments which are particular to this group^6^. According to their chemical structure, pigments of red algae have been broadly grouped into main categories as follows: closed tetrapyrroles (such as chlorophyll-a and chlorophyll-d), open tetrapyrrols (e.g., phycobilipigments), and carotenoids (e.g., polyisoprenoids)^6^. Because of their significant biotechnological potential, they have been used in numerous commercial applications and have registered patents. These pigments also exist in many colors and have been effectively used throughout history in daily life. A few instances worth mentioning include food, paper, and textile industries, water technology, and agricultural practices^4^. Interestingly, pigments exhibit advantageous biological activity as antioxidants and anticancer agents, as well as several key features that make them suitable in these various industrial contexts^4^. Therefore, they have a significant potential to meet current market needs, which increasingly focus on the health and biotechnology sectors in search of natural substances having positive effects on human health. However, there are still issues with optimizing and then scaling up extraction and purification processes, particularly when taking into account the needs for both quantity and quality from an industrial and commercial perspective^4^.

In particular, the red alga *Grateloupia gibbesii* was newly introduced to the Egyptian coast of the Mediterranean Sea^7^. Currently, this species has been identified as a synonym of *Phyllymenia gibbesii*^8^. Many studies are focused on the alien species in the Mediterranean Sea, which cause many impacts on the introducing area, such as changes in biodiversity, competition between these alien species and native ones^9^. In addition, some of them have the potential to be detrimental to the ecosystem and to other macroalgal species. Thus, it would be of interest to find out valuable substances in the domain of health and nutrition from the biomass of alien species in order to compensate the expense of eradication and as a safe and eco-friendly solution to the problem of introduction.

The basic target of the current study is to investigate several biological activities of different pigments detected in the newly recorded alga *Phyllymenia gibbesii* and to assess the concentration of these pigments.

## Results and discussion

Macroalgae are a significant basis for therapeutically valuable compounds. They are regarded as a source of unique types of biologically active substances used in several domains due to their diversity and chemical differences. Herein, the antioxidant, anti-inflammatory, hemolytic, and antibacterial properties of *P. gibbesii* acetone extract were evaluated. Its pigments were also examined to further clarify the chemical constituents.

### HPLC chromatographic analysis

**Fig. 1:**
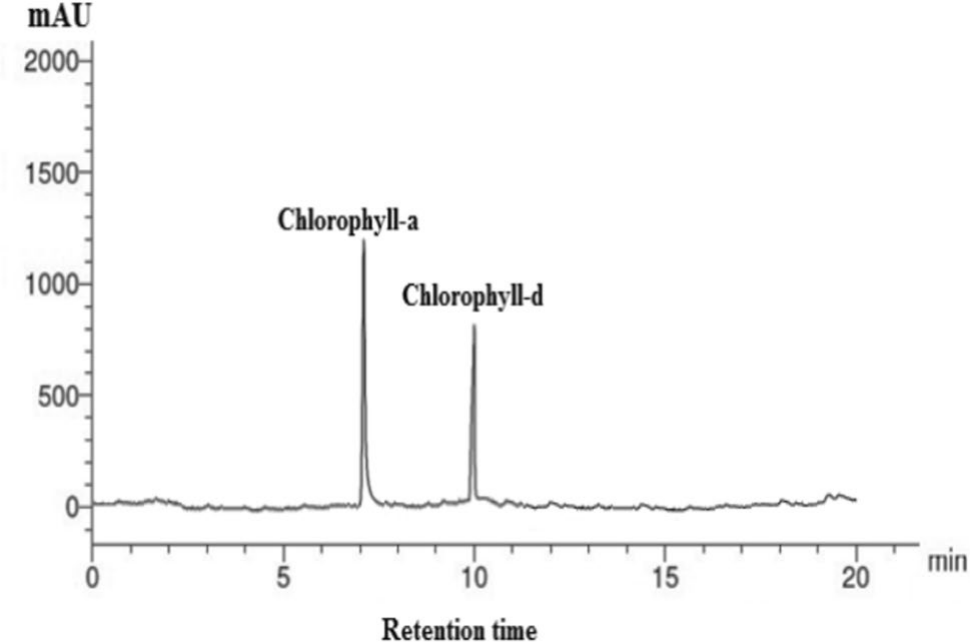
HPLC analysis of chlorophyll-a and chlorophyll-d in *P.gibbesii* acetone extract.

**Fig. 2:**
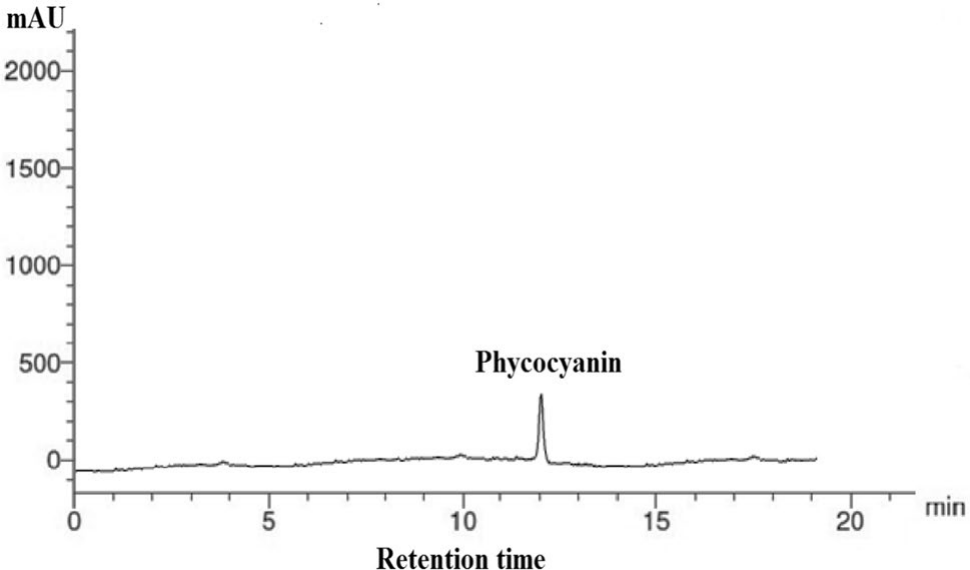
HPLC analysis of phycocyanin in *P.gibbesii* acetone extract.

**Fig. 3:**
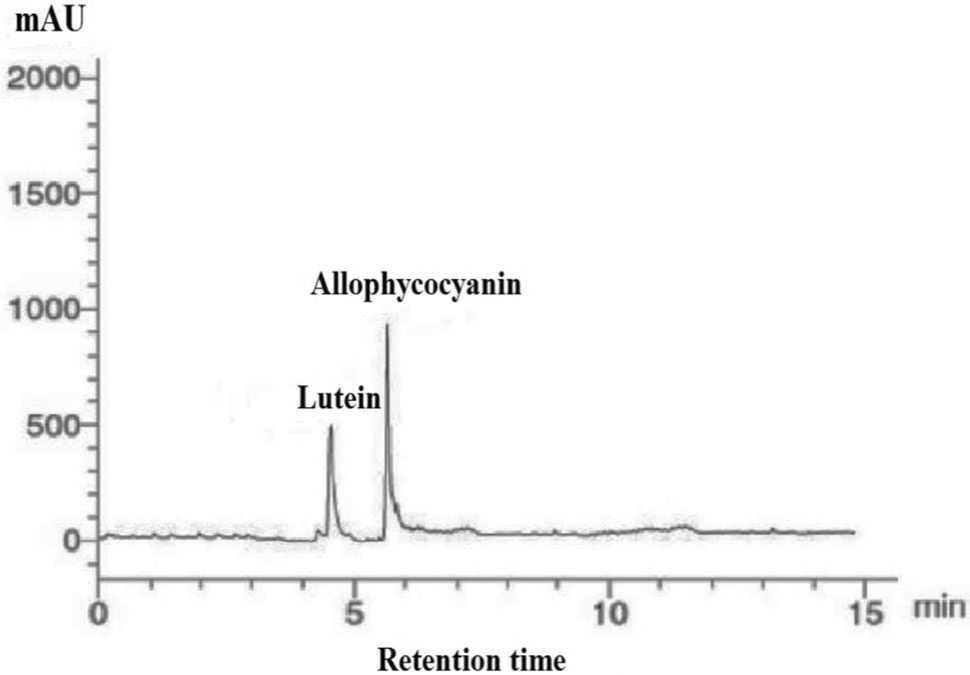
HPLC analysis of allophycocyanin and lutein in *P. gibbesii* acetone extract.

**Fig. 4:**
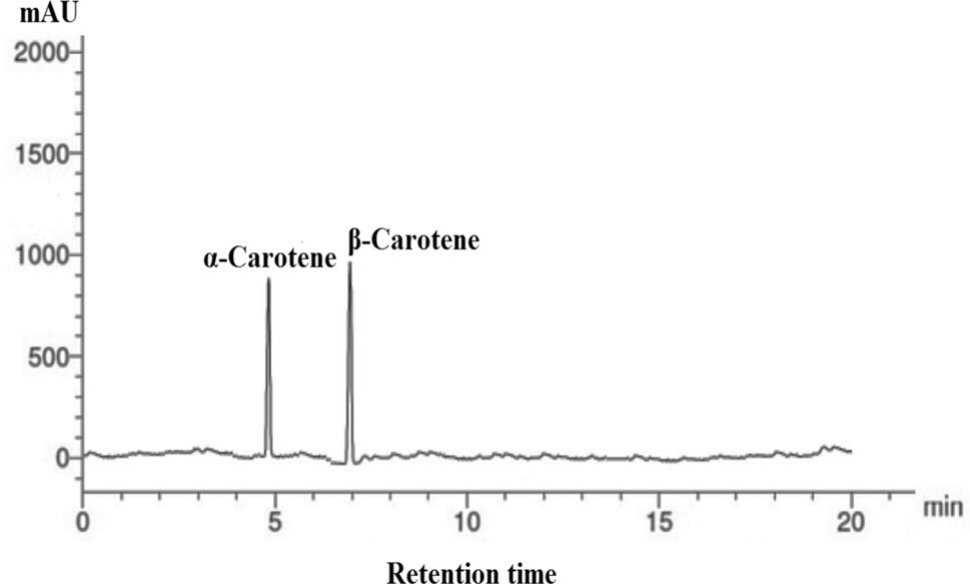
HPLC analysis of α-Carotene and β-Carotene in *P. gibbesii* acetone extract.

**Fig. 5:**
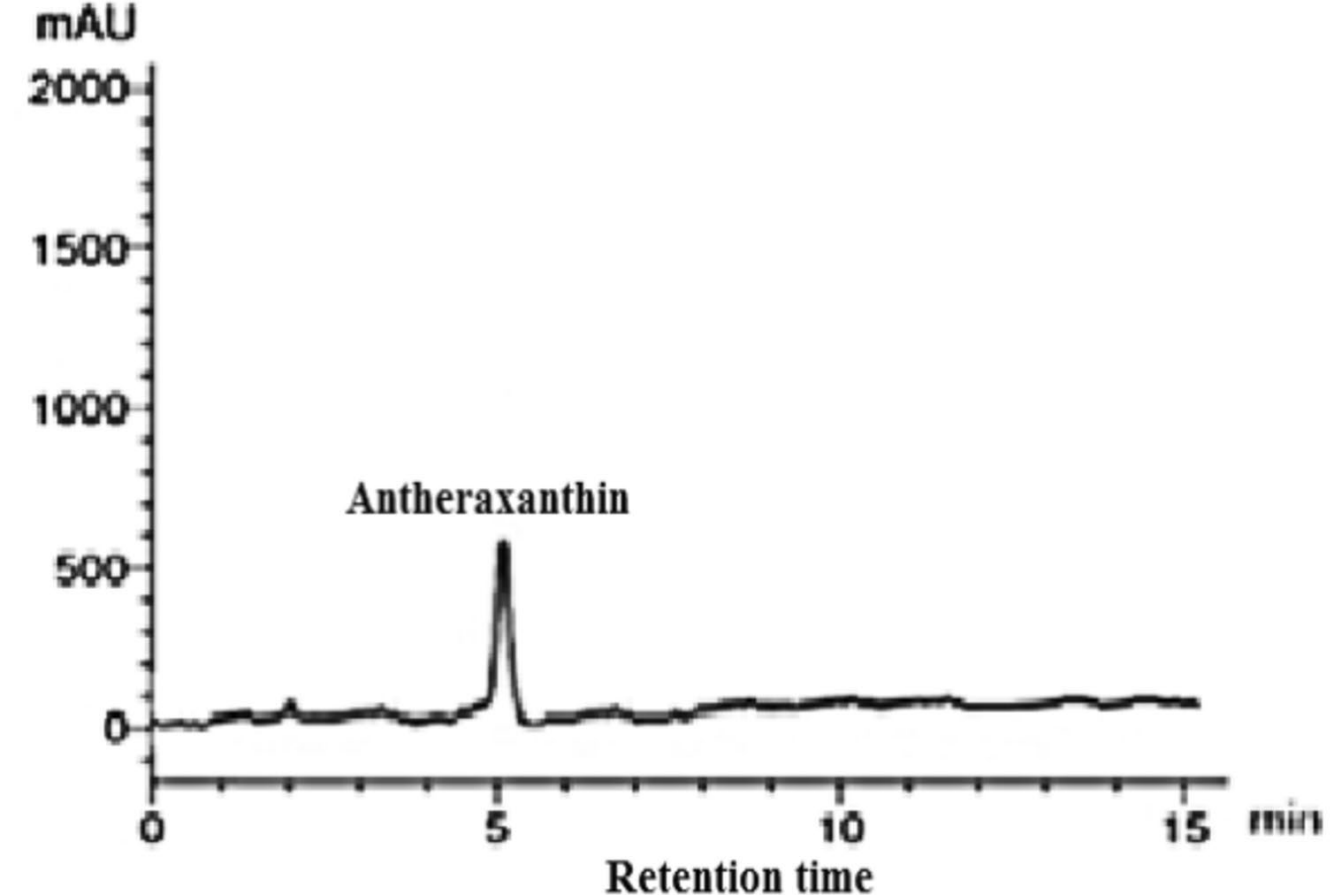
HPLC analysis of antheraxanthinin *P. gibbesii* acetone extract.

**Fig. 6:**
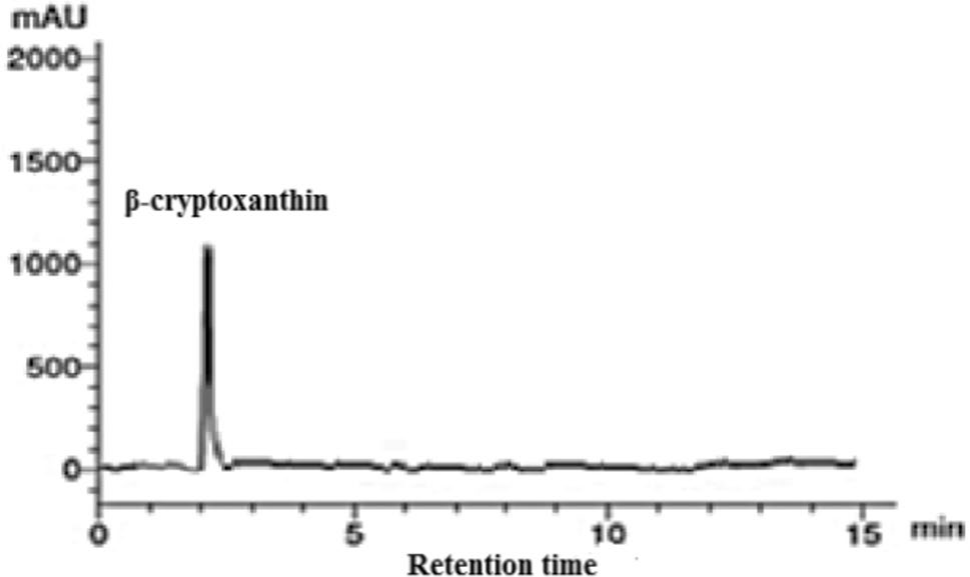
HPLC analysis of β-cryptoxanthin in *P. gibbesii* acetone extract.

**Fig. 7:**
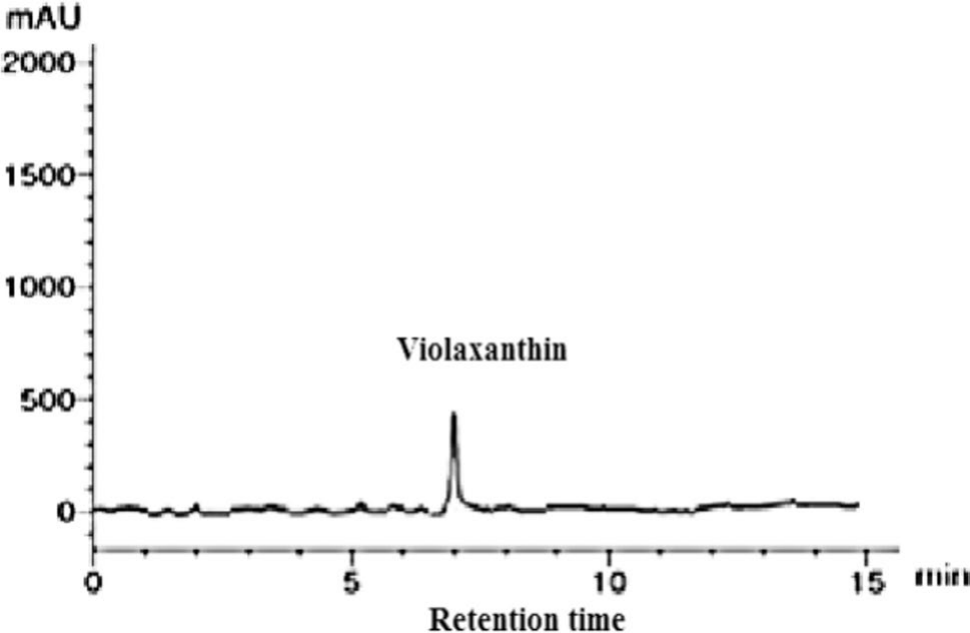
HPLC analysis of violaxanthin in *P. gibbesii* acetone extract.

To identify the pigments contents in *P. gibbesii* acetone extract*,* HPLC has been used. The *P. gibesii* chromatograms showed 10 individual photosynthetic pigments: chlorophyll-a, chlorophyll-d, phycocyanin, allophycocyanin, α-carotene and β-carotene, antheraxanthin, β-cryptoxanthin, lutein, and violaxanthin (Figs. 1–7). The two pigments; phycoerythrin and zeaxanthin, were not detected by the HPLC. The chromatogram showed that chlorophyll-d was one of the characteristic chlorophylls of *P. gibbesii*, with a lower concentration of 25.95 μg/g than that of chlorophyll-a (41.65 μg/g) (Table 1). Among the phycobiliproteins (PBP), allophycocyanin (APC) was the dominant pigment (41.95 μg/g), while phycocyanin (PC) attained only half of APC (20.60 μg/g) (Table 1).

**Table 1:** Pigments content of *P. gibbesii* acetone extract (μg/g) analyzed by HPLC.

Pigments	Pigments concentration (μg/g)
Chlorophylls:	
Chlorophyll-a	41.65
Chlorophyll-d	25.95
Total chlorophylls	67.60
Phycobiliproteins:	
Phycocyanin	20.60
Phycoerythrin	Not detected
Allophycocyanin	41.95
Total phycobiliproteins	62.55
Carotenoids:	
α-Carotene	76.10
β-Carotene	80.60
Total carotenes	156.70
Xanthophylls:	
Antheraxanthin	19.00
β-cryptoxanthin	42.50
Lutein	31.30
Violaxanthin	7.50
Zeaxanthin	Not detected
Total xanthophylls	100.30
Total carotenoids	257.00

The carotenes were the most representative pigments in the carotenoids class. The concentration of β-carotene (80.60 μg/g) was comparable to α-carotene (76.10 μg/g). Their total concentration (156.70 μg/g) exceeded that of the total chlorophylls (67.60 μg/g) and total phycobiliproteins (62.55 μg/g) (Table 1). Concerning the xanthophylls, the second group in the carotenoids, they were represented by four pigments; antheraxanthin, β-cryptoxanthin, lutein, and violaxanthin, with concentrations of 19.00, 42.50, 31.30, and 7.50 μg/g, respectively. However, the total carotenoids were the dominant class in the pigments’ profile, attaining a concentration of 257 μg/g (Table 1).

Because of a substance known as sodium-copper-chlorophyllin, chlorophyll has anti-cancer and antioxidant properties. Supplementation with chlorophyllin before meals has been proven to diminish the harm caused by aflatoxin, a highly carcinogenic mold that grows on some foods^10^. In high-risk populations, the use of chlorophyllin may aid in the prevention of liver cancer caused by aflatoxin. Moreover, chlorophyll metabolites, like phytanic acid, can be used to treat and prevent diabetes^11^. Phytanic acid also has an effect on the expression of genes related to glucose metabolism via different natural peroxisome proliferator-activated receptor isoforms^11^.

According to Dasgupta^12^, chlorophyll-a and chlorophyll-d are two of the pigments of red algae. In contrast, Kato and his co-workers^13^ mentioned that red seaweeds have only chlorophyll-a. However, the detection of chlorophyll-d is dependent on the choice of solvent for the extraction process^14^. In this trend, Osório and his co-workers^15^ investigated the red alga *Porphyra sp.* for pigment content by using a variety of solvents. Their findings indicated that acetone was the most suitable solvent for the detection of chlorophyll-d, resulting in a maximum of 16.25 μg/g, while it was absent using the N-dimethyl formamide solvent. In the present study, using acetone as solvent gave the opportunity to determine chlorophyll-d in *P. gibbesii* (25.95 μg/g) and chlorophyll-a (41.65 μg/g). In contrast to our study, Paransa and his co-workers^16^ (2020) found that chlorophyll-d was higher (16.36–21.42 μg/g) than chlorophyll-a (6.14–18.32 μg/g) in *Kappaphycus alvarezii*. This may be attributed to that chlorophyll-d can support the role of chlorophyll-a in red algae^17^.

Phycobiliproteins (PBP) have many health advantages as antioxidants and free-radical scavengers^4^ besides their potent neuroprotective, anti-bacterial^18^, anti-inflammatory^19^, anti-allergic^19^, antitumoral^19^, anti-ageing^19^, anti-Alzheimer^18^, immunomodulatory^18^, and hypocholesterolemic agents^18^. Basically, phycobiliproteins (PBP) are classified into four pigment families according to their absorption properties. Red algae comprise phycoerythrin (PE), phycocyanin (PC), and allophycocyanin (APC), while phycoerythrocyanin (PEC) is absent in this group^4^. Actually, PBP have been found to be vital in proliferating and adapting the red algae by maximizing the capabilities of algal light-harvesting in the deep layers of the water column^4^. In the present study, PE was not detected by the HPLC. This may be attributed to the methodologies of extraction of the PE. In the present study, the extraction process was based on adding acetone as a solvent to the dry powder alga, followed by HPLC for separation and isolation of pigments, while in many previous different studies, methodologies were used to determine PE based mainly on using different buffer solutions such as phosphate buffer^19^, acetic-acid sodium acetate buffer^20^, and EDTA^21^.The extraction was followed by purification, using many efficient methods like precipitation with ammonium sulfate^22^, gel filtration and ion-exchange chromatography^23^, expanded bed absorption and ion-exchange chromatography^24^.

Considering the other two pigments, allophycocyanin content was higher (41.95 μg/g) than that of phycocyanin (20.60 μg/g) in *P. gibbesii*. Francavilla and his co-workers^20^ reported that PC was the less abundant phycobiliprotein in *Gracilaria gracilis*, which is consistent with our results. Pina and his co-workers^25^ showed that the concentration of PC in *Chondrus crispus* was 149 μg/gdw, while Sukwong and his co-workers^26^ isolated from *Gelidium amansii* 56 μg/g dw of PC. These differences in PC and APC contents compared with the previous studies may be due to many factors, such as the aforementioned methodologies for extraction, the species, the time of the year when harvesting, or environmental conditions such as light intensity^27^, nutrient cycles, and photoperiod^27^.

Carotenoids in red algae are divided into carotenes (β-carotene and α carotenes) and the major xanthophylls (antheraxanthin, β-cryptoxanthin, lutein, violaxanthin, and zeaxanthin)^28^. Schöbel^29^ reported that rhodophytes lack distinctive carotenoid profiles due to either the existence or absence of specific carotenoids, which are constituents in the xanthophyll group. On the other hand, Mimuro & Akimoto^30^ noted that the existence of particular biosynthetic routes or the distribution of carotenoids with different molecular structures can be considered an index for algae classification. In this trend, Shubert and his co-workers^6^ estimated the carotenoid content of 65 subtropical red algal species from many taxonomic orders and families. The findings demonstrated that there were three main carotenoid profiles identified, with the exception of the Ceramiales and Corallinales. Shubert and his co-workers^6^ reported that most of the species had lutein as the major xanthophylls (first profile), while zeaxanthin was the major one in some other species (second profile). The third profile (Xanthophyll Cycle -related carotenoids) showed that violaxanthin or antheraxanthin were also detected in some species. In addition to these major xanthophylls, there are minor ones that depend on the interconversion of the major pigments^31^.

The variation in these trace pigments may be the reason for the unclear relationship between the carotenoid content of red algae and their phylogenetics^32^. The findings of Takaichi^33^ synergized with the previous study of Czeczuga^32^, where he reported in five red algal species belonging to different families different profiles of carotenoids than that of Shubert and his co-workers^6^. Czeczuga^32^ explained the different pigment profiles on the basis of the presence of additional minor xanthophylls such as γ-carotene, α-cryptoxanthin, lutein epoxide, mutatoxanthin, fucoxanthin, neoxanthin, and others. Our results are in agreement with those of Czeczuga^32^ and Takaichi^33^ and disagree with those of Shubert and his co-workers^6^. It is also noteworthy to mention that the taxonomic position of the *Grateloupia*, *Phyllymenia*, and *Prionitis* genera remains controversial, and many species are still re-identified or transferred from one family to another. Currently, the species *G. gibbesii* is considered a synonym of *P. gibbesii*^8^, which was transferred to the genus *Phyllymenia* based on the female reproductive structures and other attributes^8^. Thus, it is necessary to screen again the pigment profiles of the members of order Halymeniales, which was previously included in order Cryptomeniales and has recently undergone many taxonomic changes and may show different pigment profiles.

In the present study, the profile of *P. gibbesii* showed that β-cryptoxanthin was the principle xanthophyll component (42.50 μg/g) forming 42.37%, followed by lutein (31.30 μg/g) forming 31.21% of total carotenoids, with antheraxanthin and violaxanthin forming collectively 26.42% and with the absence of zeaxanthin. On the other hand, β-carotene and α carotenes were comparable in *P. gibbesii* forming (80.6 μg/g) and (76.1 μg/g), contributing 31.36% and 29.61% of total carotenoids, respectively. Thus, the percentage of total carotenes (60.97%) was higher than that of xanthophylls (39.03%). However, the content of the two carotenes is dependent on the lycopene pathway, whether it is mainly cyclized into either β-carotene, or α-carotene or both^33^. Also, the differences between algal species and their geographical distribution, seasonality, environmental conditions, and light regime are controlling factors of carotenoids content^34^. In the present study, total carotenoids content was higher (257.00 μg/g) than chlorophyll-a (41.65 μg/g).This is consistent with the findings of Barsanti & Gualtieri^35^, who reported that algal species exposed to high light intensity typically exhibit a relatively high amount of carotenoid pigments in comparison with chlorophyll-a.

### Total phenolics and flavonoids contents (TFC)

The acetone extract of *P. gibbesii* had a total content of phenolics of 146.67 ± 0.61 mg/g and a total content of flavonoid of 104.40 ± 0.10 mg/g (Table 2).

**Table 2:** Total phenolics and total flavonoids in *P. gibbesii.*

Concentration	Total phenolics (mg GAE/g)	Total flavonoids (mg CAT/g)
Extract of *P. gibbesii*	146.67 ± 0.61	104.40 ± 0.10

Numerous biological actions, like antimicrobial, anti-diabetic, antioxidant, and anticancer capabilities, are provided by phenolic compounds, which include one or more phenolic rings that may be halogenated^36^. The main classes of phenolics in the red algae are bromophenols, terpenoids, phenyl propanoid derivatives, polymerized hydroxycinnamyl alcohols, and mycosporine-like amino acids^37^. In the current study, phenolic content in *P. gibbesii* was much higher (146.67 ± 0.61mgGAE/g) than that of Shabaka & Moawad^38^ in the same alga during the same season (May, 2019) at the same site (Eastern Harbor) utilizing different solvents (chloroform, ethyl acetate, and distilled water), resulting in 4.745, 6.629 and 3.211 mgGAE/g, respectively. The great difference between the phenolic content of the two algal samples can be attributed to the site of algal collection and its environmental conditions, algae growth stage, sample preparation, and the method used for determination of the phenolic content, in addition to the choice of the extracting solvent**,** where the acetone in our study showed very high efficiency compared to the other solvents.

The significance of total flavonoid content is mostly due to its redox properties, that could explain its antioxidant, anti-inflammatory, and antibacterial properties towards many microbes^39^. In addition, the antioxidant property of the flavonoid content is attributed to its hydroxyl group structuring, which may be responsible for capturing and stabilizing much more free radicals^39^. However, in the current study, flavonoid content in *P. gibbesii* was much higher** (**104.40 ± 0.10 mg CAT/g**)** than that of Khristi and his co-workers^40^ in *G. indica* (0.0241QUE mg/g) using methanol as extracting solvent, while Ruiz-Medina and his co-workers^5^ found that flavonoid content was 1.44 mg CAE/g in *G. imbricata* using 80% methanol. This may be attributed to using different methodologies and different solvents and other limitations like species of algae, harvesting season, and environmental conditions^37^.

### Biological activities of *P.gibbesii* extract

The results of the biological activities of the newly recorded alga *P. gibbesii* are shown in (Tables 3–5).

**Table 3:** Antioxidant, hemolytic and anti-inflammatory activities (%) of *P. gibbesii* extract.

Concentration (μg/mL)	Radical scavenging activity		Hemolytic activity (%)	Anti-inflammatory activity (%)
	DPPH %	ABTS %		
10	50.62	40.31	18.75	81.25
20	56.44	45.93	29.38	70.63
40	64.36	56.54	36.00	64.00
80	70.92	79.32	44.00	56.00
100	81.44	89.62	49.88	50.13

**Table 4:** Inhibitory concentrations (IC_50_) (μg/mL), at which the algal extract showed antioxidant, hemolytic and anti-inflammatory activities.

Concentration (μg/mL)	Radical scavenging activity (μg/mL)		Hemolytic activity (μg/mL)	Anti-inflammatory activity (μg/mL)
	DPPH	ABTS		
Standard material	Ascorbic acid	Ascorbic acid	Aspirin	Diclofenac
IC_50_ of standard	3.44 ± 0.01	7.73 ± 0.01	5.94 ± 0.01	331.24 ± 0.04
IC_50_ of *P. gibbesii*	9.88 ± 0.01	21.77 ± 0.01	100.25 ± 0.01	99.75 ± 0.05

**Table 5:** Antimicrobial activity of acetone extract of *P. gibbesii*.

Microbe	Gramstain and shape	Concentration (mg/mL)/inhibition zone diameter (mm)			
		0.025	0.05	0.1	MIC
*K.pneumonia* ATCC700603	Gram −ve, rod-shaped	11	15	18	0.025
*E. coli*ATCC25922	Gram −ve, rod-shaped	20	26	30	0.025
*S. aureus* ATCC25923	Gram + ve, spherically-shaped	ND	11	18	0.050
*S. pyogenes*EMCC1772	Gram + ve, spherically-shaped	ND	11	15	0.050
*C.albicans*EMCC105	Yeast	19	21	28	0.025

MIC is abbreviated to minimum inhibition concentration (mg/mL).

*ND* not detected.

**Diameter includes 5 mm well diameter.

### Antioxidant activity

The DPPH was a significant metric for assessing the antioxidant activity of an extract. Also, the ABTS assay has been extensively used to test the ability of different extracts, fractions, and/or pure substances to scavenge free radicals.

### Activity of DPPH radical scavenging

The results indicated that increasing the extract concentration boosted the DPPH’^s^ capacity (Table 3). Knowingly, the better the antioxidant properties, the smaller the value of IC_50_. Indeed, it was 9.88 ± 0.01 g/mL for the *P. gibbesii*, while it was 3.44 ± 0.01 g/mL for the positive control (L-ascorbic acid) (Table4).

### ABTS radical scavenging assay

The results demonstrated that, like the DPPH, increasing the concentration of the acetone extract of *P. gibbesii* enhanced the scavenging activity scored by the ABTS assay (Table 3). Moreover, the *P. gibbesii* acetone extract showed ABTS activity with an IC_50_ value of 21.77 ± 0.01 μg/mL, corresponding to7.73 ± 0.01 μg/mL for L-ascorbic acid (Table 4).

Antioxidants are efficacious in safeguarding cells against ROS-mediated oxidative damage, particularly under unfavorable conditions^41^. Several synthetic antioxidants are commercially available, but due to safety concerns, there has been a lot of interest in switching to natural plant-based antioxidants^41^. The current data showed high DPPH radical scavenging activity in *P. gibbesii*, with a maximum of 81.44%, which was higher than that of *G. indica*, showing a maximum of 69.90%^40^. Similarly, the ABTS assay showed high activity, with a maximum of 89.62%. Noticeably, the free radical scavenging capacity increased with increasing the concentration of the algal extract. This scavenging capacity may be due to the hydrogen-donating ability of the antioxidant properties of *P. gibbesii*^42^. This might be interpreted by the view that there is a positive correlation between pigment concentration and the antioxidant activity of the algae species. Increasing pigment production is induced by increasing light intensity, which in turn boosts antioxidant activity^28^. Furthermore, according to Zhang and his co-workers^43^, the antioxidant activity of seaweeds may be influenced by the presence of flavonoids and polyphenols components. However, their concentrations affected the activity potential.

### Hemolytic activity assay

The hemolytic activity of *P. gibbesii* increased gradually with the increase in the algal extract concentrations (10, 20, 40, 80, and 100 μg/mL), attaining a maximum capacity of 49.88% (Table 3). The hemolytic activity exhibited a higher IC_50_ value of 100.25 ± 0.01 μg/mL compared with that of aspirin (5.94 ± 0.01 μg/mL) (Table 4).

Hemolysins are lipid and protein substances that cause the erythrocyte to burst and release hemoglobin, harming a number of vital organs, including the heart, liver, and kidney^44^. Moreover, hemolysis may result in anemia or jaundice. As a result, while testing the biological activities of algal extracts and derivatives for pharmaceutical purposes, it is necessary to investigate the hemolytic activity^45^.

Herein, the hemolytic potency of the algal extract was higher (IC_50_ = 100.25 ± 0.01 μg/mL) than that of aspirin, which represents a positive control (IC_50_ = 5.94 ± 0.01 μg/mL).On contrast, the *P. gibbesii* hemolytic activity was lower than that of the red alga *Hypnea cornuta* extract (IC_50_ = 1396.23 μg/mL), the green alga *Ulva prolifera* (IC_50_ = 1298.25 μg/mL) and the brown alga *Turbinaria triquetra* (IC_50_ = 973.68 μg/mL)^46^. This showed that *P. gibbesii* extract is better than that of these species for pharmaceutical purposes.

### *Invitro* anti-inflammatory activity

The anti-inflammatory activity of *P. gibbesii* extract at different concentrations (10, 20, 40, 80, and 100 μg/mL) is represented in (Table 3). This extract exhibited a lower IC_50_ value of 99.75 ± 0.05 μg/mL compared with that of the commercial Diclofenac (331.24 ± 0.04 μg/mL) (Table 4). The development of many severe diseases has been linked to complicated biochemical events that distinguish the inflammation process^2^. The anti-inflammatory activity of the extracts obtained from different red algae species has been confirmed by several studies (e.g., Chen and his co-workers^47^). Within the current study, theIC_50_ value of the algal extract (99.75 ± 0.05 μg/mL) was much lower than that of the commercial diclofenac (331.24 ± 0.04 μg/mL). This showed that *P. gibbesii* is of great potential to develop natural sources of antioxidants that may help in combating inflammatory diseases.

### Antimicrobial activity

All of the examined microorganisms were susceptible to the effects of *P. gibbesii* acetone extract, particularly at the highest concentration (0.1 mg/mL) (Table 5). The extract had the greatest impact on the Gramme-negative bacterium *E. coli* and the yeast *C. albicans*.

Nowadays, the bioactive compounds derived from seaweed metabolites have been documented as antiviral, antibacterial, antifungal, and antiprotozoal agents^48^. Abu-Ghannam & Rajauria^49^ explained the antimicrobial mechanism in view of carotenoids role, which causes the accumulation of the immunological enzyme lysozyme that breaks down bacterial cell walls. The antimicrobial activity of *P. gibbesii* acetone extract was promising, as all the tested microorganisms were inhibited to varying degrees. This was confirmed by the minimum inhibitory concentration (MIC) (0.025 mg/L), which showed the low resistance and the high sensitivity of these microorganisms to the algal extract, particularly *E. coli* and *C. albicans*. Moreover, the efficiency of the algal extract augmented with the high concentration. In this trend, Abdel-Latif and his co-workers^50^ carried out a study on *Grateloupia doryphora*, which exhibited a wide range of antimicrobial action towards the investigated microbial species, namely *Bacillus subtilis, Enterococcus faecalis, Staphylococcus aureus, E. coli, Pseudomonas aeruginosa,* and *C. albicans*. Obviously, *P. aeruginosa* was the most affected microbe, with higher inhibition zones (30, 28, and 27 mm) for methanolic, ethanolic extracts and ethyl acetate, respectively. In addition, Negm and his co-workers^51^ prepared silver nanoparticles from *Grateloupia* sp., showing activity versus *E. coli, S. faecalis*, *S. aureus*, *P. aeruginosa*, and *V. damsela*. Moreover, Hajri and his co-workers^52^ studied the antibacterial, among other biological activities, of the aqueous extract of *Grateloupia sparsa* in the formula of cobalt oxide nanoparticles (Co_3_O_4_NPs). Amazingly, their data showed that Co_3_O_4_NPs had stronger antibacterial activity than Ciprofloxacin, a common antibiotic.

## Conclusion

Recently, the red alga *P. gibesii* was recorded in the Mediterranean Sea of the Egyptian coast. Herein, it was investigated for phytochemical content and pigments’ profiles. The *P. gibesii* chromatograms showed ten individual photosynthetic pigments: chlorophyll-a, chlorophyll-d, α-carotene, β-carotene, phycocyanin, allophycocyanin, antheraxanthin, β-cryptoxanthin, lutein, and violaxanthin. The results of *P. gibbesii* acetone extract showed many biological activities, including antioxidant, anti-inflammatory, and antimicrobial activity, which confirmed their potential biochemical components. As a result, the alga might be seen as a potential source of bioactive substances that can be applied in several industries, especially pharmaceutical, biotechnological, and nutraceutical. This gives the opportunity to recycle this alien species in a safe and eco-friendly manner.

## Materials and methods

### Area of the study

The algal samples of the newly recorded *P. gibbesii* were collected from the Eastern Harbor, Alexandria, Egypt, during spring (May, 2019). The algal samples were collected at a depth of 0.5–1 m at the Scout Club, which is situated in the Eastern Harbor at 31°13.3`N latitude and 29°53.10`E longitude.

### Sample collection

The algal samples were manually collected, then rinsed in seawater at the site of collection to get rid of the adherent sediments and other impurities before being placed in clean plastic bags. The samples were kept in an ice box at 4 °C. Upon arrival at the lab, algal samples were promptly rinsed with tap water to remove any remaining epiphytes and contaminants. Rodrguez-Prieto and his coworkers^7^ collected the species from the Eastern Harbor during the same period of the current study (May–June, 2019) and identified the newly introduced alga as *Grateloupia gibbesii* Harvey by using the rbcL gene and provide a detailed description of the morphology, anatomy and in details the pre-and post-fertilization stages of the female reproductive structures. The phylogeny based on the chloroplast-encoded rbcL gene of *Grateloupia* sensu lato clusters the *Grateloupia* samples from Alexandria, Egypt, with a single sequence of *G. gibbesii* Harvey from Charleston, South Carolina. The species belongs to the Class Florideophyceae, Order Halymeniales, Family Grateloupiaceae, and is currently considered to be the synonym of *Phyllymenia gibbesii* (Harvey)^8^.

About 250 g of the alga was air dried to a consistent weight at ambient temperature, and then the algal sample was chopped into tiny chunks and ground to a fine powder for the next procedures.

### Preparation of algal extract

A weight of 100 g of algal powder was macerated in 400 mL of acetone for 48 h. In a rotating evaporator (Stuart RE300), the acetone was filtered and concentrated at 40 °C under pressure. The acetone extract was then stored at −20 °C for further analysis^53^.

### High-performance liquid chromatography (HPLC)

Three groups of pigments were measured using the HPLC technique. They are, namely, chlorophylls (chlorophyll-a, and chlorophyll-d), phycobiliproteins (allophycocyanin, phycocyanin, and phycoerythrine), and carotenoids (α-carotene, β-carotene, antheraxanthin, lutein, β-cryptoxanthin, violaxanthin, and zeaxanthin).

High-pressure liquid chromatography was used to analyze chlorophyll-a, and chlorophyll-d under isocratic conditions on the instrument (Agilent 1100 Series):C18 column with a diode array detector set at detection wavelengths (λdet.); 430 and 660 nm. The mobile phase was acetonitrile/methanol/ethyl-acetate in the ratio of 60:20:20, with a flow rate; 1 mL/min, and temperature of 25 °C.

Phycobiliproteins (phycoerythrin and phycocyanin) were analyzed by HPLC by using a UV detector set at detection wavelengths: 260 nm under isocratic conditions, which included the following: mobile phase- acetonitrile/methanol in the ratio of 80:20, respectively. The flow rate was 1 mL/min at a temperature of 25 °C.

Allophycocyanin and lutein were analyzed using an HPLC system with an Echromatech Mod.C1620, column 18, by using a detector set at 620 nm. The isocratic conditions were a Na-phosphate buffer (pH 3.0) as the solvent (A) and NaCl and methanol as the solvents (B) in the ratio of 45:55, respectively. The flow rate of the process was 0.5 mL/min at a temperature of 25 °C.

Carotenoids (α-carotene, β-carotene, and zeaxanthin) were assessed using an HPLC with a diode array detector and data collected by a Chemstation. Sample (20 mL) was loaded on an ODS-1 reversed phase column (250′4.6 mm i.d., 5 mm particle size). Acetonitrile, 2-propanol, and water were eluted isocratically in the following proportions: 39:57:4. The detection wavelength was 450 nm, while the detection flow rate was 1 mL/min at a temperature of 25 °C.

Antheraxanthin analysis was carried out by HPLC analysis using the system echromatechMod.C1620, column C18, with a detector set at detection wavelengths of 490 nm, and under isocratic conditions, with the mobile phase made up of methanol, water, acetonitrile, and dichloromethane in the ratio of 70:4:13:13. The temperature was 25 °C, and the flow rate was 1 mL/min. The conditions were the same for β-cryptoxanthin, except a detector set at 450 nm was used, and the mobile phase was methanol/acetone in the ratio of 70: 30. The process detected violaxanthin using a 450 nm detector under isocratic conditions with mobile phase (A) acetonitrile/water in a ratio of 90:10 and mobile phase (B) ethyl acetate. The flow rate was 1 mL/min, and the temperature was 25 °C.

Different standards for each pigment were utilized to prepare its calibration curve for quantification. All standards were bought from Sigma and were 95% pure (HPLC grade). The retention times and spectral data were compared to those of the authentic standards in order to identify the HPLC peaks.

### Determination of total phenolic content (TPC)

According to Dewanto et al.^54^, the Folin-Ciocalteu reagent was used to determine the total phenolic content. 200 μL of the dissolved sample (one mg of extract after being dissolved in one mL of deionized water) was added to 600 μL of distilled water and 100 μL of Folin-Ciocalteu reagent. Before 2% Na_2_CO_3_ was added, the mixture was agitated and allowed to stand for 6 min. A final volume of 3 mL was adjusted with distilled water, which was then well mixed. The absorbance at 650 nm was measured in comparison to the blank produced after 30 min of incubation in the dark. A standard curve was produced utilizing various gallic acid concentrations (standard, 0–100 μg /mL). Based on the calibration curve, the following linear equation was used to compute the total phenol content (TPC), which was expressed as gallic acid equivalent (GAE)/mg of dry weight: y = 0.0095x–0.0409 & R^2^ = 0.9953. Where x is the concentration (GAE mg/g extract), y is absorbance, and R^2^ is the correlation coefficient.

### Determination of total flavonoid content (TFC)

Total flavonoids of the algal extract were estimated using a modified colorimetric method published by Sakanaka et al.^55^ Catechol was used as a standard at concentrations of 20–200 μg /mL. Precisely, 250 μL of extract or standard solutions were added to 1.25 mL of distilled water, 75 μL of 5% sodium nitrite solution (NaNO_2_), and 150 μL of 10% aluminium chloride solution (AlCl_3_) five minutes later. 0.6 mL of distilled water and 0.5 mL of 1 M sodium hydroxide (NaOH) were added after 6 min. Afterward, the mixture was shaken well, and the absorbance was determined at 510 nm. Based on the calibration curve, the following linear equation was used to assess total flavonoid content (CE): Y = 0.0079 x−0.109 & R^2^ = 0.986. Where R^2^ is the correlation coefficient, X is the concentration (CE mg/g extract), and y is absorbance.

### Assays determining antioxidant activity

#### DPPH radical scavenging activity

The 2, 2-diphenyl-1-picrylhydrazyl (DPPH) technique, developed by Brand-Williams et al.^56^, was modified to test the algal extract's capacity to scavenge free radicals. One milliliter of the radical solution (0.2 Mm DPPH in methanol, 0.0078 g/100 mL) was added to 1 mL of the sample, or standard solution, at various concentrations (1:1, v/v).The mixture was incubated at room temperature in the dark for 30 min, and the absorbance was detected at 517 nm. To create a calibration curve, the ascorbic acid solution was employed as the standard with a concentration range of 5–200 μg/mL. However, DPPH radical scavenging activity was qualified as mg ascorbic acid equivalent (AAE)/g dried sample. Using the following equation, the percentage of DPPH radical-scavenging activity was determined:

$$ {\text{DPPH radical scavenging activity }}(\% {\text{ inhibition}}) = \frac{{\left( {Abs_{Control} - Abs_{Sample} } \right)}}{{Abs_{Control} }} \times 100 $$

All reagents were added to the control sample, with the exception of the algal extract. All measurements were made in triplicate, and the average result was tabulated.

#### ABTS radical scavenging assay

The assay of 2,2′-Azino-bis (3-ethylbenzothiazoline-6-sulfonic acid; ABTS) was used to determine the ABTS radical scavenging activity^57^. The extract’s ability to scavenge ABTS was compared with that of dibutyl hydroxytoluene (BHT), and inhibition% was calculated as follows:

$$ {\text{ABTS radical scavenging activity }}\left( \% \right)\, = \,[\left( {{\text{Abs}}.{\text{control}} - {\text{Abs}}.{\text{sample}}} \right)\left] {/\left( {{\text{Abs}}.{\text{control}}} \right)} \right]\, \times \,{1}00 $$

Where, Abs. sample is the absorbance of ABTS radical + sample extract/standard, whereas Abs. control is the absorbance of ABTS radical + methanol.

#### Hemolytic activity assay

The experiment was carried out in 2 mL microtubes via a modified method of Farias et al.^58^. First, a two-fold serial dilution of each extract with 0.9% NaCl between 1000 and 1.9 μg/mL was made and set aside. Afterward, 900 μL of each extract dilution were placed in a new microtube, and 100 μL of a 1% red blood cell suspension (A, B, and O human blood types or rabbit blood) were added. The microtube was then incubated at 37 °C for an hour. All tubes were centrifuged for 5 min at 3,000 × g. A 96-well plate containing the supernatant (200 μL) was connected to a microplate reader to measure different absorbances at 540 nm. The cell suspensions of each human blood type (100 μL) were mixed with distilled water or 0.9% NaCl (900 μL). The following is how the percentage of hemolysis was determined: ${\text{Hemolysis}}\% \, = {\text{ Abs}}_{test} /{\text{Abs}}_{pc} \times { 1}00$.

Where Abs_pc_ = Abs 540 of the 1% cell suspension treated with distilled water and Abs_test_ = Abs 540 of the 1% cell suspension treated with a sample test.

#### Anti-inflammatory activity assay

Using a few minor adjustments, the human red blood corpuscles (HRBCs) membrane stabilizing method^59^ was used to evaluate the extracts’ anti-inflammatory activity in vitro. Blood was drawn from a healthy volunteer without using any inflammatory medications for the 14 days prior to such an experiment. The blood was then transferred to heparinized centrifuge tubes and spun at a speed of 3000 rpm. After being cleaned with isosaline, the packed cells were converted into a 10% suspension in regular saline. As a reference, diclofenac potassium (50 μg/mL) was employed. About 5 mL of the reaction mixture includes 1 mL of 0.15 M phosphate buffer (pH 7.4), 1 mL of test solution (1 mg/mL) in normal saline, and 0.5 mL of 10% HRBC in normal saline, together with 2 mL of hypotonic saline (0.25% NaCl). One molesters of isotonic saline was adjusted as the control instead of the test solution. The mixtures were incubated for 30 min at 56 ºC, cooled under running water, and then centrifuged for 20 min at 3000 rpm. Using a visible spectrophotometer, the supernatant's absorbance at 560 nm was measured. The experiment was carried out three times. 100% of lyses are represented by the control. The following calculation was used to compute the percentage of membrane stabilization: Protection% = 100−(OD of test/OD of control) × 100.

By graphing the percentage of inhibition with respect to the control versus the treatment concentration, the extract/drug concentration for 50% inhibition (IC_50_) was revealed.

#### Antimicrobial activity assay

Reference microbial species, including *Klebsiella pneumonia* ATCC700603 and *Escherichia coli* ATCC25922, were used as Gram negative bacteria. In addition, *Staphylococcus aureus* ATCC25923 and *Streptococcus pyogenes* EMCC1772 were used as Gram-positive bacteria. Moreover, one yeast strain (*Candida albicans* EMCC105) was used. For these pathogens, the antimicrobial effect of *P. gibbesii* extract was evaluated.

The yeast strain was cultivated in potato dextrose broth at 28 °C for 24 h, while the bacterial strains were grown in nutritional broth in g/L at 37 °C. A sterile 100 mm diameter Petri plate was then filled with 100 μL of the inoculums (1 × 10^8^ cfu/mL) and inculcated on nutrient agar medium in g/L. The well-cut diffusion method was applied to detect the antibacterial activity^60^. The wells were prepared in the plates by utilizing a cork-borer (0.5 cm) in nutrient agar plates inoculated with the reference microbes. The algal extract was added with a volume of 100 μL to each well. All tested bacteria were incubated for 24 h at 37 °C, while *C. albicans* EMCC 105 was incubated at 28 °C for 3–5 days. To ascertain the minimum inhibitory concentration (MIC), *P. gibbesii* extract was used at three concentrations (0.025, 0.05, and 0.1 mg/mL). The diameter of the inhibition zone surrounding the well (mm) was measured in three triplicates, and the average values were estimated.

## Data Availability

During this study, all obtained or analyzed data are included in this published article.
